# A Novel Combination Treatment with Honokiol and Rapamycin Effectively Restricts c-Met-Induced Growth of Renal Cancer Cells, and also Inhibits the Expression of Tumor Cell PD-L1 Involved in Immune Escape

**DOI:** 10.3390/cancers12071782

**Published:** 2020-07-03

**Authors:** Akash Sabarwal, Samik Chakraborty, Simran Mahanta, Selina Banerjee, Murugabaskar Balan, Soumitro Pal

**Affiliations:** 1 Division of Nephrology, Boston Children’s Hospital, Boston, MA 02115, USA; akash.sabarwal@childrens.harvard.edu (A.S.); samik.chakraborty@childrens.harvard.edu (S.C.); msimran155@gmail.com (S.M.); banerjeeselina1@gmail.com (S.B.); murugabaskar.balan@childrens.harvard.edu (M.B.); 2 Harvard Medical School, Boston, MA 02115, USA

**Keywords:** receptor tyrosine kinase, renal cell carcinoma, c-Met, Honokiol, mTOR inhibitor, PD-L1

## Abstract

The mTOR inhibitor Rapamycin has tumor inhibitory properties; and it is also used as an immunosuppressive agent after organ transplantation. However, prolonged Rapamycin treatment re-activates Akt and can promote cancer growth. Honokiol is a natural compound with both anti-tumorigenic and anti-inflammatory properties. Here, we assessed the anti-tumor effects of Rapamycin and Honokiol combination in renal cell carcinoma (RCC). Receptor tyrosine kinase c-Met-mediated signaling plays a major role in RCC growth. We observed that compared with Rapamycin alone, Rapamycin + Honokiol combination can effectively down-regulate c-Met-induced Akt phosphorylation in renal cancer cells; and it markedly inhibited Ras activation and cell proliferation and promoted G1 phase cell cycle arrest. The combination treatment significantly induced ROS generation and cancer cell apoptosis even when c-Met is activated. Importantly, Honokiol, but not Rapamycin, decreased c-Met-induced expression of the co-inhibitory molecule PD-L1, implied in the immune escape of renal cancer cells. In mouse renal cancer cells and Balb/c splenocytes co-culture assay, Rapamycin + Honokiol markedly potentiated immune-cell-mediated killing of cancer cells, possibly through the down-regulation of PD-L1. Together, Honokiol can effectively overcome the limitation of Rapamycin treatment alone; and the combination treatment can markedly restrict the growth of RCC, with particular importance to post-transplantation renal cancer.

## 1. Introduction

There are very limited treatment options for advanced renal cell carcinoma (RCC). The five-year survival rate for patients with metastatic RCC is less than 10% as the tumors get highly resistant to standard chemo- and radiotherapy [1]. Although different therapeutic agents, including, multiple kinase inhibitors (like, sorafenib, sunitinib, cabozantinib and others), mammalian target of Rapamycin (mTOR )inhibitors, and some immune checkpoint inhibitors have been suggested as first-line treatment for advanced RCC, the treatment responses are not long-standing; and the tumors progress due to drug resistance and immune escape [2,3,4]. Thus, the identification of novel therapeutic target(s)/combination therapy for RCC is urgently needed.

The development and rapid progression of cancer is a major problem in immunosuppressed patients, like patients receiving an organ transplant [5,6]. Interestingly, renal cancer is one of the most common cancers in transplant patients [7,8,9,10]. Although immunosuppressive therapies (used in transplant patients) have reduced acute allograft rejection, they are also associated with increased risk of cancer [11]. Classically, following immunosuppressive therapy, such as with calcineurin inhibitor (CNI), the immune surveillance mechanism gets impaired [12]. However, apart from classical function, immunosuppressive agents can also directly regulate other pro-tumorigenic signaling pathways to promote tumor growth. We identified that CNI can activate the proto-oncogene ras, and promote a rapid progression of renal cancer [13]. We have recently reported that the receptor tyrosine kinase, c-Met (mesenchymal-epithelial transition factor), which is over-expressed in renal cancer, promotes the survival of renal tumor cells through the regulation of the anti-oxidant cytoprotective molecule heme oxygenase-1 (HO-1); c-Met also modulates the expression of the negative co-stimulatory molecule, PD-L1 (Programmed Death-Ligand 1), involved in immune escape of renal cancer cells [14]. c-Met is a known inducer of Ras, which is hyper-active in renal cancer; and Ras has cross-talk with mTOR. Interestingly, we found that the c-Met pathway can be activated in renal cancer cells following CNI treatment [13].

The mTOR inhibitor rapamycin (RAPA) is often used in transplant patients to prevent organ rejection through the down-regulation of T cell proliferation [15]. Interestingly, it has anti-tumorigenic potential to reduce tumor angiogenesis, and is used for the treatment of renal cancer to prolong progression-free survival [16]. However, the RAPA responses are short lived, and most of the patients finally develop drug resistance. Also, prolonged RAPA treatment relieves the negative feedback loop for Akt activation through the inhibition of S6-kinase (S6K); and thus, it can promote renal tumor growth through PI-3K-mTOR activation [17,18]. In addition, prolonged RAPA treatment is also associated with proteinuria and other major complications. Thus, the RAPA treatment alone cannot prevent the growth and progression of post-transplantation cancer, and new therapeutic combinations are needed.

Honokiol (C_18_H_18_O_2_), a biphenolic natural product originally isolated from Magnolia obovate, is a promising agent for mediating anti-inflammatory, pro-apoptotic and chemopreventive functions [19,20,21]. Honokiol can promote tumor cell apoptosis. Honokiol-induced apoptosis can be mediated through the activation of caspases, and the induction of pro-apoptotic Bax and Bad. Honokiol also suppresses Ras-mediated activation of phospholipase D, and induces cyclophilin D to promote mitochondrial permeability transition pore-associated cell death. We have demonstrated that Honokiol can effectively down-regulate CNI-induced activation of the Ras-Raf pathway in renal cancer cells [13]. We also found that Honokiol promotes apoptosis by inhibiting CNI-induced Nrf2/HO-1, which plays a major role in Ras-mediated survival of renal cancer cells [22]. It has been suggested that Honokiol can modulate the redox state of cancer cells by regulating the level of reactive oxygen species (ROS) [23,24,25]. Honokiol, is being tested in pre-clinical models for its anti-tumorigenic potential. In addition, due to its anti-inflammatory property [26], Honokiol can also be utilized for the treatment of transplant patients to sustain their immune suppression, and to reduce the dose of other immunosuppressive agents. 

New treatment options need to be explored for post-transplantation renal cancer. The mTOR inhibitor RAPA is used for the treatment of transplant patients, and it has anti-angiogenic properties. However, as discussed above, it cannot prevent post-transplantation cancer due to several major limitations. Honokiol, which has both anti-inflammatory and anti-tumorigenic potential, can be a unique and very promising therapeutic agent (when used in combination therapy with RAPA) to prevent post-transplantation renal cancer. In the present study, we show that that the combination treatment with RAPA and Honokiol effectively restricts the growth and cell cycle progression of renal cancer cells by inhibiting the c-Met-HO-1 axis; it also down-regulates the expression of PD-L1, which plays a major role in the immune escape of renal cancer cells. Thus, this combination therapy can have a great potential to prevent/restrict renal cancer in both immunosuppressed as well as other RCC patients.

## 2. Results

### 2.1. A Combination Treatment with RAPA and Honokiol Effectively Down-Regulates c-Met-Induced Phosphorylation of Akt, mTOR and S6 in Renal Cancer Cells

**Figure 1:**
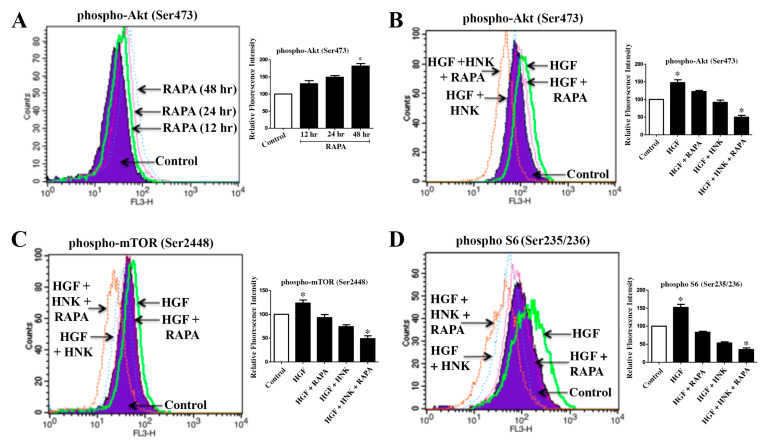
Rapamycin (RAPA) + Honokiol (HNK) combination downregulates HGF-induced phosphorylation of Akt, mTOR and S6 in renal cancer cells. (**A**) The 786-0 cells were treated with either RAPA (15 μM) or vehicle for 12–48 h. Following treatment, intracellular phospho-Akt (Ser473) expression (in permeabilized cells) was analyzed by flow cytometry. (**B**–**D**) Serum starved 786-0 cells were pre-treated with combinations of RAPA (15 μM) and HNK (40 μM) for 4 h, and then incubated with either HGF (50 ng/mL) or vehicle alone for 1 h. Following treatment, cells were analyzed for the intracellular levels of phospho-Akt (Ser473), phospho-mTOR (Ser2448) and phospho-S6 (Ser235/236) by flow cytometry. (**A**–**D**), representative data of three independent experiments; and the bar graphs presented next to histogram overlays represent the quantification of changes in relative fluorescence intensities compared with the control (control values were considered as 100%). The *columns* represent the mean ± S.D. of duplicate experimental readings. * *p* < 0.05 compared with respective controls.

The mTOR inhibitor RAPA is used in transplant patients to prevent allograft rejection; however, as discussed earlier, prolonged RAPA treatment can promote Akt activation through the withdrawal of negative feedback loop, and thus can facilitate tumor growth [17]. Here, we first checked the effect of RAPA treatment on Akt phosphorylation (Ser473) in 786-0 renal cancer cells. We observed that the prolonged treatment with RAPA increased Akt phosphorylation in a time-dependent manner (Figure 1A). The treatment with the c-Met ligand, Hepatocyte Growth Factor (HGF) (a known inducer of the tumor-promoting pathways in renal cancer), also increased Akt phosphorylation (Figure 1B). Next, we studied the effect of Honokiol (HNK) on HGF-induced Akt phosphorylation in renal cancer cells; and how is it altered in the presence of RAPA. As shown in Figure 1B, Honokiol down-regulated HGF-induced Akt phosphorylation up to the basal level; however, a combination of RAPA and Honokiol significantly decreased HGF-induced Akt phosphorylation below the basal level. c-Met is a potent inducer of the mTOR pathway, which is hyper active in renal cancer [14]. Next, we evaluated the effect of RAPA + Honokiol combination treatment on HGF-induced phosphorylation of mTOR (Ser2448) and S6 protein (Ser235/236). We found that the combination of RAPA and Honokiol markedly down-regulated HGF-induced phosphorylation of both mTOR and S6 below the basal level (Figure 1C,D). We also checked the effect of RAPA and Honokiol combination in the absence of HGF treatment. We observed that the RAPA + Honokiol combination treatment markedly down-regulated the level of phospho-Akt, phospho-mTOR and phospho-S6 in renal cancer cells; however, there was no significant change in the total protein levels (Supplementary Figure S1A). We found similar observations in both 786-0 and ACHN (Supplementary Figure S1B) renal cancer cells. Together, our findings suggests that RAPA + Honokiol combination treatment can be a novel therapeutic option for renal cancer, with particular importance to post-transplantation cancer; and Honokiol can overcome the limitation of prolonged RAPA treatment, and restrict RAPA-induced Akt activation. 

### 2.2. RAPA and Honokiol Inhibits Renal Cancer Cell Proliferation, Down-Regulates Active Ras, and Induces G1 Phase Cell Cycle Arrest

**Figure 2:**
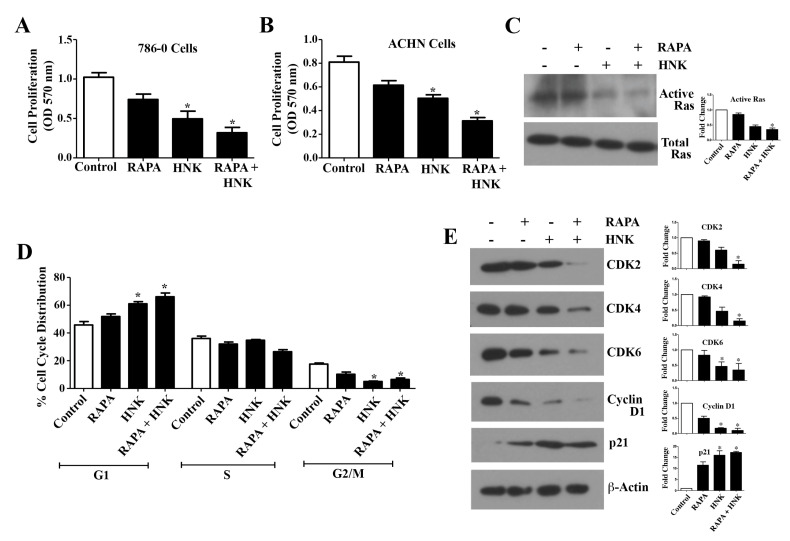
Combination treatment with Rapamycin (RAPA) and Honokiol (HNK) effectively inhibits renal cancer cell proliferation and induces cell cycle arrest. (**A**) 786-0 and (**B**), ACHN cells were treated with RAPA (15 μM) and Honokiol (40 μM) either alone or in combination for 48 h and cell proliferation was measured by MTT assay. (**C**) 786-0 cells were treated with RAPA (15 μM) and Honokiol (40 μM) either alone or in combination for 1 h. Following treatment, cell lysates were used to assess Ras activation statuses using GTP-bound Ras pull down assay kit, as described in “Materials and Methods” section. (**D**) 786-0 cells were treated with RAPA (15 μM) and Honokiol (40 μM) either alone or in combination for 24 h. Following treatment, cells were stained with propidium iodide and the percentage of cells in different phases of the cell cycle was determined by flow cytometry and the quantifications are presented. (**E**) Following treatment as described in D, 786-0 cells were lysed and the expression of CDK2, CDK4, CDK6, Cyclin D1, p21 and β-actin were determined by Western blot analysis. A, B, and D, the *columns* represent the mean ± S.D. of triplicate readings of two different samples. * *p* < 0.05 compared with respective controls. (**C**,**E**) results shown are representative of three independent experiments; and the bar graphs presented next to the Western blots correspond to the fold changes in the expression of the indicated proteins, which were calculated by densitometric analysis of the intensities of protein bands normalized to those of β-actin. The control values were considered as 1 fold. The *columns* represent the mean ± S.D. of the readings of two different samples. * *p* < 0.05 compared with respective controls.

Here, we first checked how the RAPA + Honokiol combination treatment can regulate renal cancer cell proliferation. As shown in Figure 2A,B, in both 786-0 and ACHN cells, RAPA + Honokiol combination significantly decreased the cell proliferation compared with vehicle-treated controls. There was also some decrease in the proliferation of normal renal proximal tubular epithelial cells (RPTEC) following RAPA + Honokiol treatment (Supplementary Figure S2A). As active Ras is a key player in regulating growth-promoting signals in renal cancer [27], we checked the status of Ras activation in the treated cells. Although the RAPA treatment alone did not change the level of active GTP-bound Ras, the combination of RAPA + Honokiol markedly decreased active Ras; but there was no change in the level of total Ras (Figure 2C and Supplementary Figure S2B).

As abrupt cell cycle regulation is important for cancer progression, we next checked the effect of RAPA and Honokiol on cell cycle distribution of renal cancer cells. We found that Honokiol caused G1 phase arrest of renal cancer cells compared with controls; and in combination with RAPA, it further increased the G1 phase cell cycle population (Figure 2D). Next, we analyzed the molecular markers for the G1 phase of cell cycle. As shown in Figure 2E and Supplementary Figure S2C, we found that RAPA did not affect the levels of CDK2 and CDK4, while it did decrease the expression of CDK6 and Cyclin D1 and increased the expression of an important cyclin dependent kinase inhibitor (CDKI), p21. HNK decreased the expression of CDK2, CDK4, CDK6, Cyclin D1 and increased the expression of p21. Consistent with our previous observations, the combination of RAPA + Honokiol was more potent in decreasing the expression of all G1 phase CDKs and Cyclins, and increasing the expression of p21 (Figure 2E and Supplementary Figure S2C). Together, these results suggest that RAPA + Honokiol combination is potent in down-regulating renal cancer cell proliferation possibly through the inhibition of active Ras and induction of G1 phase cell cycle arrest.

### 2.3. RAPA and Honokiol Combination Treatment Promotes Reactive Oxygen Species (ROS) Generation, and Induces Apoptosis of Renal Cancer Cells

**Figure 3:**
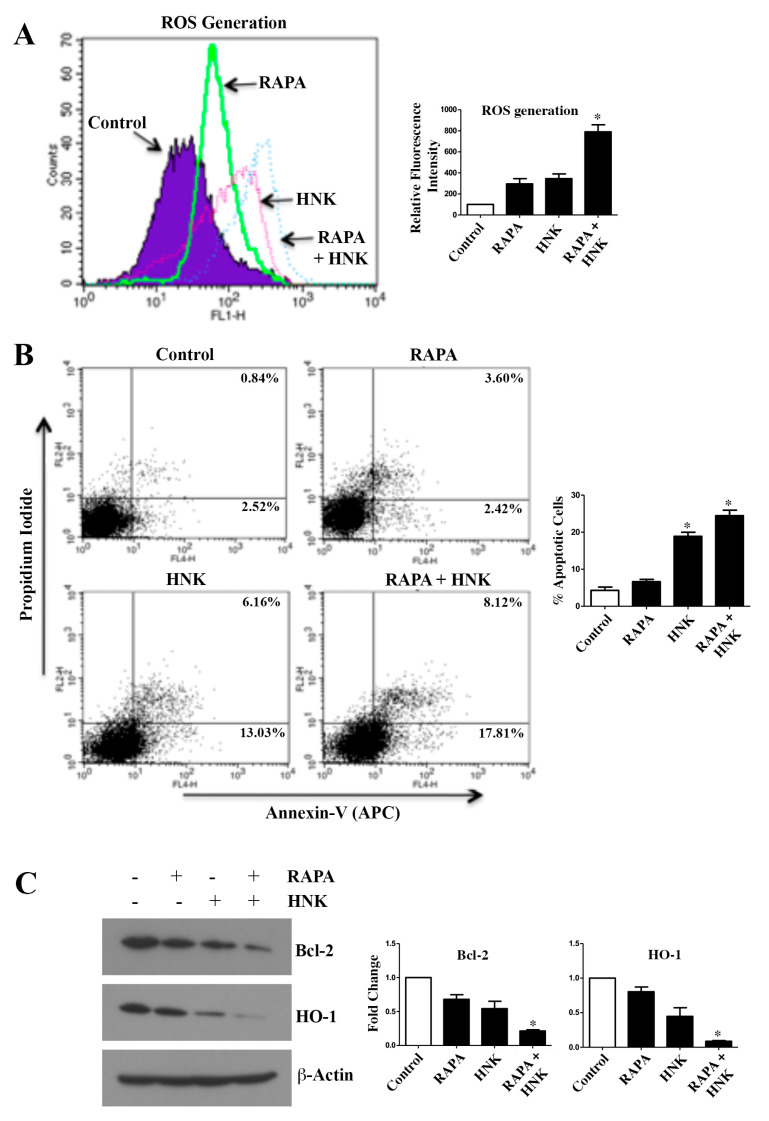
Honokiol (HNK) + Rapamycin (RAPA) combination markedly induces ROS generation and promotes renal cancer cell apoptosis. (**A**) 786-0 cells were treated with RAPA (15 μM) and Honokiol (40 μM) either alone or in combination for 6 h. Following treatment, cells were stained with 2′,7′-dichlorofluorescein diacetate-based total ROS detection agent and cellular ROS levels were assessed by flow cytometry. (**B**) 786-0 cells were treated with RAPA (15 μM) and Honokiol (40 μM) either alone or in combination for 48 h. Following treatment, cells were stained with annexin V (APC) and propidium iodide and the apoptotic indices were analyzed by flow cytometry. (**C**) 786-0 cells were treated as described in (**B**), and cell lysates were used to measure the expression of Bcl-2, HO-1 and β-actin by Western blot. (**A**–**C**), results shown are representative of three independent experiments. (**A**) the bar graph presented next to histogram overlay represents the quantification of change in relative fluorescence intensities compared with the control (control values were considered as 100%). The *columns* represent the mean ± S.D. of duplicate experimental readings. (**B**) the bar graph presented next to the FACS (Fluorescence-activated cell sorting) analysis, represents the percentage of total (early + late) apoptotic cells. The *columns* represent the mean ± S.D of duplicate experimental readings. (**C**) the bar graphs presented next to the Western blots correspond to the fold change in the expression of the indicated proteins, which were calculated by performing densitometric analysis of the intensities of protein bands normalized to those of β-actin. The control values were considered as 1 fold. The *columns* represent the mean ± S.D of two different samples. In (**A**–**C**) * *p* < 0.05 compared with respective controls.

Many chemotherapeutic agents induce high levels of cellular reactive oxygen species (ROS), which can cause oxidative damage to macromolecules leading to the induction of apoptosis [28]. Thus, we investigated the effect of RAPA and Honokiol on ROS generation in renal cancer cells. As shown in Figure 3A, we found that RAPA treatment increased ROS compared with control cells; but HNK was more potent in increasing the ROS levels. However, the combinatorial treatment with RAPA + Honokiol significantly increased the ROS generation. Next, we checked the status of cellular apoptosis following RAPA and Honokiol treatment. Through apoptosis assay (Figure 3B), we found that although Honokiol treatment increased renal cancer cell apoptosis, RAPA alone slightly promoted cancer cell death compared with control. However, RAPA + Honokiol combination treatment markedly induced cellular apoptosis; the total apoptotic cells (early + late) increased from (2.52 + 0.84) = 3.36% (control cells) to (17.81 + 8.12) = 25.93% (RAPA- + HNK-treated cells). We found a similar observation in ACHN cells (Supplementary Figure S3A). Also, there was a modest increase in cellular apoptosis in normal renal epithelial cells (RPTEC) following RAPA + Honokiol treatment (Supplementary Figure S3B). We checked the status of anti-apoptotic marker, Bcl-2 and the cytoprotective ROS scavenger molecule, HO-1 in renal cancer cells following treatments. The combination treatment with RAPA + Honokiol markedly decreased the expression of both Bcl-2 and HO-1 (Figure 3C). Thus, RAPA + Honokiol can act as a potent combination therapy to promote renal cancer cell death.

### 2.4. RAPA and Honokiol Treatment Effectively Restricts c-Met-Induced Survival of Renal Cancer Cells

**Figure 4:**
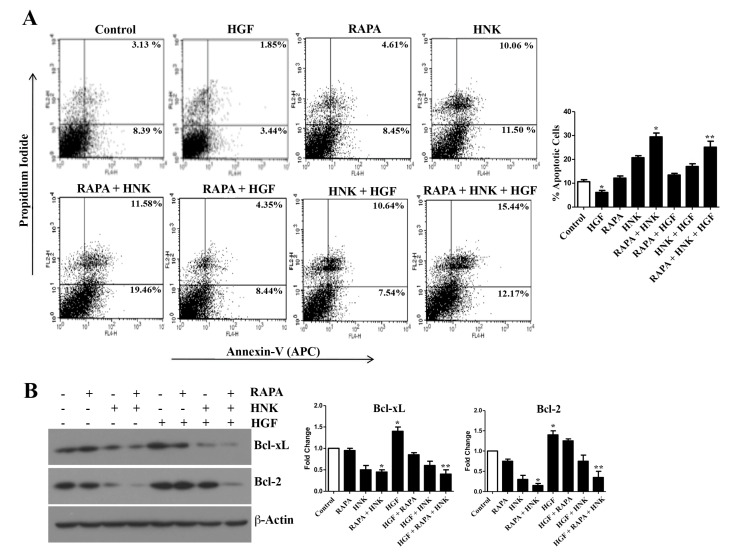
Honokiol (HNK) down-regulates HGF-mediated anti-apoptotic signals and promotes renal cancer cell apoptosis. (**A**) 786-0 cells were treated with RAPA (15 μM) and Honokiol (40 μM) either alone or in combination, and incubated in the presence or absence of HGF for 48 h. Following treatment, cells were stained with annexin V (APC) and propidium iodide and the apoptotic indices were analyzed by flow cytometry. (**B**) Cell lysates from (**A**) were used and the expression of Bcl-2, Bcl-xL and β-actin were assessed by Western blot. (**A**,**B**), data shown are representative of three independent experiments. (**A**), the bar graph next to the FACS analysis represents the percentage of total (early + late) apoptotic cells. The *columns* represent the mean ± S.D. of duplicate experimental readings. * *p* < 0.05 compared with control and ** *p* < 0.05 compared with HGF-treated cells. (**B**), the bar graphs presented next to the Western blots correspond to the fold change in the expression of the indicated proteins, which were calculated by performing densitometric analysis of the intensities of protein bands normalized to those of β-actin. The control values were considered as 1 fold. The *columns* represent the mean ± S.D of two different samples. * *p* < 0.05 compared with control and ** *p* < 0.05 compared to HGF-treated cells.

As mentioned before, the c-Met-induced pathway promotes the growth and survival of renal cancer cells and mediates chemoresistance [29]. We wanted to study if RAPA and Honokiol combination can restrict c-Met-induced renal cancer cell growth following treatment with the c-Met ligand HGF. As shown in Figure 4A and Supplementary Figure S4, we observed that HGF decreased the cellular apoptosis in control as well as Honokiol-treated cells. However, RAPA + Honokiol combination treatment markedly increased renal cancer cell apoptosis even in the presence of HGF. The total apoptotic cells (early + late) increased from (8.39 + 3.13) = 11.52% (control cells) to (12.17 + 15.44) = 27.61% (RAPA- + HNK- + HGF-treated cells). We checked the expression status of anti-apoptotic markers (Bcl-xL and Bcl-2) in these cells (Figure 4B). HGF treatment increased the basal expression of Bcl-xL and Bcl-2. Although both basal Bcl-xL and Bcl-2 were down-regulated with Honokiol, the expression of Bcl-2 was again increased in Honokiol-treated cells in the presence of HGF. However, the combination treatment with RAPA + Honokiol markedly down-regulated both Bcl-xL and Bcl-2 even in the presence of HGF. Thus, RAPA + Honokiol combination can effectively restrict c-Met-induced growth of renal cancer cells.

### 2.5. The Efficiency of RAPA and Honokiol to Induce Tumor Cell Apoptosis is Increased in c-Met Knockout Cells

**Figure 5:**
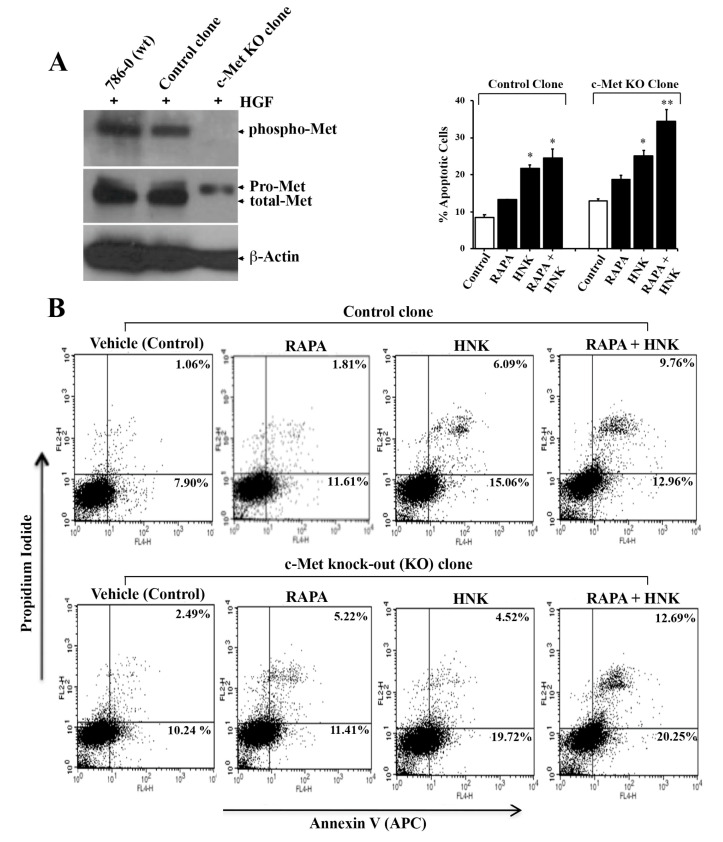
Enhanced apoptotic effects of Honokiol (HNK) in c-Met knock-out renal cancer cells. (**A**) 786-0 (wild type), CRISPR/Cas9-mediated c-Met knocked-out clonal 786-0 cells (c-Met KO clone) and control clonal cells were treated with HGF (50 ng/mL) for 1 h. Following treatment, cell lysates were utilized and levels of phospho-Met (Tyr1234/1235), pro-Met/total-Met and β-actin were analyzed by Western blot. (**B**) Control and c-Met KO clones were treated with RAPA (15 μM) and Honokiol (40 μM) either alone or in combination and in the presence of HGF (50 ng/mL) or vehicle for 24 h. Following treatment, cells were stained with annexin V (APC) and propidium iodide and the apoptotic indices were analyzed by flow cytometry. (**A**,**B**), data shown are representative of three independent experiments; and the bar graph (above *B*, the FACS analysis) represents the percentage of total (early + late) apoptotic cells. The *columns* represent the mean ± S.D. of duplicate experimental readings. * *p* < 0.05 compared with respective controls; and ** *p* < 0.05 compared with RAPA- + HNK-treated cells in control clones.

Here, we wanted to study if the RAPA + Honokiol combination is more effective to induce apoptosis in c-Met knockout renal cancer cells. We have generated stable c-Met knockout renal cancer cells using CRISPR/Cas9 (Clustered Regularly Interspaced Short Palindromic Repeats)/(CRISPR associated protein 9) gene editing technique (Figure 5A). We confirmed the knock-down of c-Met in these stable cells compared with control clones. Interestingly, we have observed that the combination treatment with RAPA and Honokiol markedly increased tumor cell apoptosis in c-Met knockout (KO) clones compared with control clones (Figure 5B). The combination treatment increased the total (early + late) apoptotic cells from (12.96 + 9.76) = 22.72% (in control clone) to (20.25 + 12.69) = 32.94% (in c-Met KO clone). Thus, although the RAPA + Honokiol combination treatment is effective to promote tumor cell apoptosis in wild type cells (where c-Met is active, control clones), its efficiency is increased following c-Met knock down (c-Met KO clones). 

### 2.6. The c-Met-Induced PD-L1 Expression on Renal Cancer Cells is Down-Regulated by RAPA and Honokiol Combination Treatment

**Figure 6:**
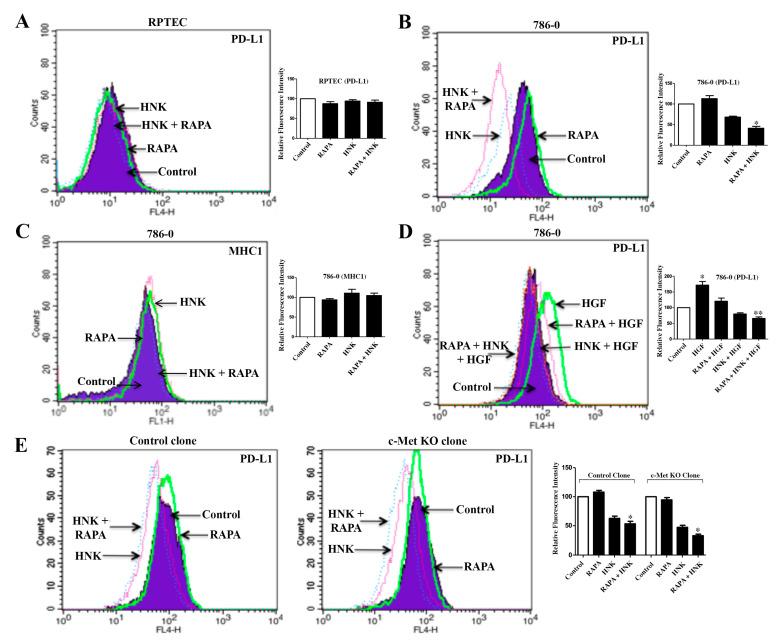
Honokiol (HNK) + Rapamycin (RAPA) combination decreases c-Met-induced PD-L1 expression in renal cancer cells. (**A**) RPTEC and (**B**–**C**), 786-0 cells were treated with RAPA (15 μM) and Honokiol (40 μM) either alone or in combination for 48h. Following treatment, cells were stained with PD-L1/MHC1 antibody and the cell surface expression of PD-L1/MHC1 were analyzed by flow cytometry. (**D**) 786-0 cells were treated with RAPA (15 μM) and Honokiol (40 μM) either alone or in combination and in the presence of HGF (50 ng/mL) or vehicle for 48 h. Following treatment, the cell surface expression of PD-L1 was analyzed by flow cytometry. (**E**) Control and c-Met KO clones (*top* and *bottom panel* respectively) were treated with RAPA (15 μM) and Honokiol (40 μM) either alone or in combination for 48 h, and the cell surface expression of PD-L1 was analyzed by flow cytometry. (**A**–**E**), data shown are representative of three independent experiments; and the bar graphs presented next to histogram overlays represent the quantification of changes in relative fluorescence intensities compared with the control (control values were considered as 100%). The *columns* represent the mean ± S.D. of duplicate experimental readings. In (**B**), * *p* < 0.05 compared with control; in (**D**), * *p* < 0.05 compared with control and ** *p* < 0.05 compared with HGF-treated cells; in (**E**), * *p* < 0.05 compared with respective controls.

PD-1 and its ligand PD-L1 are key immune checkpoint regulatory molecules that act as co-inhibitory factors to limit the T cell responses [30]. We have demonstrated that renal cancer cells express PD-L1, which can play an important role in immune escape of tumor cells; and the induction of c-Met promotes PD-L1 expression [14]. Here, we wanted to explore the effect of RAPA and Honokiol treatment on PD-L1 expression in renal cancer cells. As shown in Figure 6A, there was no significant change in basal PD-L1 in normal renal epithelial cells (RPTEC) following RAPA and Honokiol treatment. In contrast, in 786-0 renal cancer cells (where c-Met is over-expressed and can induce PD-L1), Honokiol decreased the basal PD-L1 expression, while RAPA alone did not mediate any significant change; however, the combination of RAPA and Honokiol markedly down-regulated the basal PD-L1 (Figure 6B). Interestingly, there was either slight increase or no significant change in the expression of MHC1 (Major Histocompatibility Complex (MHC) Class I) on renal cancer cells following RAPA/Honokiol treatments (Figure 6C).

As mentioned before, c-Met is either highly over-expressed or mutated in renal cancer cells compared with normal renal epithelial cells [14]. Thus, we next, we checked the effect of RAPA + Honokiol treatment on c-Met-induced PD-L1 expression in renal cancer cells. As shown in Figure 6D, HGF treatment (c-Met ligand) increased PD-L1 compared with control cells. However, RAPA + Honokiol completely blocked c-Met-induced PD-L1, and brought it to the basal level. We also examined the effect of RAPA + Honokiol on PD-L1 expression in c-Met knock-out cells. Compared with control clones, the combination treatment was more efficient in down-regulating PD-L1 in c-Met knock-out clones (Figure 6E). Together, this combination treatment can effectively inhibit c-Met-induced PD-L1 expression in renal cancer cells, and thereby may restrict their immune escape. 

### 2.7. RAPA and Honokiol Combination Treatment Promotes Immune-Mediated Apoptosis of Renal Cancer Cells, Possibly through the Down-Regulation of PD-L1

**Figure 7:**
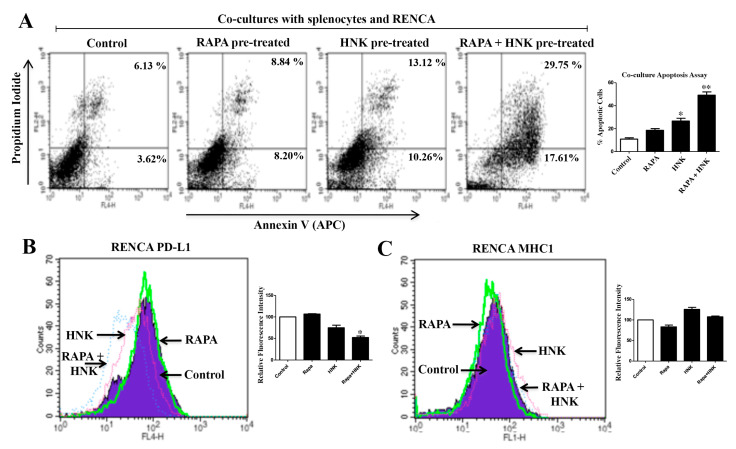
Honokiol (HNK) potentiates immune cell-mediated killing on renal cancer cells. (**A**) RENCA cells were pretreated with RAPA (15 μM) and Honokiol (40 μM) either alone or in combination for 24 h. Following treatment, freshly isolated splenocytes (from syngeneic Balb/c mouse, as described in “Materials and Methods” section) were added to establish RENCA/splenocytes co-culture for an additional 24 h. At the end of the co-culture, splenocytes that were growing in suspension were removed, and the adherent RENCA cells were collected. Then, RENCA cells were stained with annexin V (APC) and propidium iodide and the apoptotic indices were analyzed by flow cytometry. (**B**,**C**) cell surface expression of either PD-L1 or MHC1 in RENCA were analyzed by flow cytometry. (**A**–**C**) data shown are representative of three independent experiments. (**A**) the bar graph represents the percentage of total (early + late) apoptotic cells. The *columns* represent the mean ± S.D of duplicate experimental readings. * *p* < 0.05 compared with control; and ** *p* < 0.05 compared with HNK-treated cells. (**B**–**C**) the bar graphs presented next to histogram overlays represent the quantification of changes in relative fluorescence intensities compared with the control. (control values were considered as 100%) The *columns* represent the mean ± S.D. of duplicate experimental readings. * *p* < 0.05 compared with control.

Our previous experiments clearly showed that the combination treatment with RAPA + Honokiol inhibits the expression of the negative co-stimulatory molecule PD-L1 on renal cancer cells. Here, we wanted to evaluate the functional significance to know if this combination treatment can facilitate the immune-mediated killing of cancer cells. To this end, we performed a co-culture experiment utilizing the splenocytes from Balb/c mice and syngeneic renal cancer cells (RENCA) that were pre-treated with combinations of RAPA and Honokiol or the vehicle alone (control). As expected (data not shown), there was increased basal apoptosis of cancer cells when they were co-cultured with splenocytes. As shown in Figure 7A, the pre-treatments with RAPA/Honokiol increased splenocyte-mediated killing of RENCA cells compared with control; however, when the cancer cells were pre-treated with RAPA + Honokiol combination, it significantly increased splenocyte-mediated killing. The total apoptotic cells (early + late) increased from (3.62 + 6.13) = 9.75% (control RENCA) to (17.61 + 29.75) = 47.36% (RAPA- + Honokiol-pre-treated RENCA). We also checked the status of PD-L1 in co-cultured RENCA cells. We observed that the RAPA + Honokiol pre-treatment markedly decreased the expression of PD-L1 on RENCA cells (Figure 7B); however, there was no significant change in the expression of MHC1 (Figure 7C). Thus, the pre-treatment with this combination can effectively induce splenocyte-mediated apoptosis of renal cancer cells, possibly through the down-regulation of PD-L1 on cell surface.

## 3. Discussion

New therapeutic combinations are needed for the treatment of advanced renal cancer, which is also a major problem in transplant patients [10]. Honokiol is a natural compound with unique anti-inflammatory and anti-tumorigenic potential [19,21,26]. The mTOR inhibitor rapamycin (RAPA), which is being used for the treatment of both renal cancer and transplant patients, has many limitations [31]. In this study, we demonstrate that a novel combination of RAPA + Honokiol can effectively down-regulate the Ras-mTOR pathway to inhibit renal cancer cell growth; and it also inhibits PD-L1 expression to promote immune-mediated killing of renal cancer cells. In addition, our results suggest that the RAPA + Honokiol combination can act as novel therapeutics for post-transplantation renal cancer. 

RAPA, the mTOR inhibitor, is used for the treatment of renal cancer patients; it is also being used for transplant patients to prevent allograft rejection [15]. However, as mentioned before, prolonged RAPA treatment can promote Akt phosphorylation and facilitate tumor growth; it also has other limitations and tumors often develop therapeutic resistance [18]. mTOR has significant cross-talk with the Ras pathway, which is hyperactive in renal cancer cells, possibly through the induction of receptor tyrosine kinases (like, c-Met, EGFR (Epidermal Growth Factor Receptor) etc.). It is a challenge to down-regulate the hyperactive Ras in cancer cells. For this study, we first performed a cell proliferation assay to test the effect(s) of different concentrations of RAPA and Honokiol (either alone or in combinations) in renal cancer cells (Supplementary Figure S5A). For all of our experiments, we selected a minimal concentration of RAPA (15 μM) and Honokiol (40 μM), at which they inhibit ~50% cell proliferation. Our findings suggest that the combination of RAPA + Honokiol markedly inhibits the expression of active Ras in renal cancer cells. Most importantly, this combination effectively prevents c-Met-induced phosphorylation of Akt and mTOR. However, our data also suggest that c-Met may activate some other signaling pathway(s) that may not be suppressed by this combination treatment. Thus, RAPA + Honokiol can act as a unique therapeutic combination to down-regulate the hyperactive Ras-mTOR pathway in renal cancer cells. 

Cell cycle dysregulation is one of the important hallmarks of cancer cells. Many chemotherapeutic agents induce cell cycle arrest to inhibit cancer cell proliferation; and they promote apoptosis [32]. Previously, it has been demonstrated that honokiol can induce cell cycle arrest in some other cancer types [33,34,35]; however, there are no reports on Honokiol-mediated cell cycle regulations in renal cancer. Here, we observe that RAPA + Honokiol markedly induces G1 phase arrest of renal cancer cells; and it also significantly decreases their proliferation. As mentioned before, chemotherapeutic agents can kill the cancer cells through ROS generation. However, cancer cells often bypass this effect, and become chemoresistant. The cytoprotective anti-oxidant molecule Nrf2/HO-1 plays a major role in scavenging cellular ROS [36]. In our previous studies, we have demonstrated that the c-Met pathway promotes the induction of Nrf2/HO-1 in renal cancer cells; and it mediates growth-promoting signals against oxidative stresses [22]. In this study, we show that this novel combination therapy using Honokiol effectively promotes the apoptosis of renal cancer cells, possibly through the inhibition cytoprotective HO-1 and up-regulation of cellular ROS. Importantly, RAPA + Honokiol markedly increased cellular apoptosis even following the activation of c-Met. 

PD-L1 is one of the key immune-checkpoints that can play an important role in immune escape of cancer cells; and it has been reported that PD1 expressed by tumor-infiltrating immune cells is associated with poor outcome of renal cancer patients [37]. We have recently demonstrated that the activation of c-Met up-regulates PD-L1 on renal cancer cells [14]. In this study, we show that although RAPA alone does not mediate any significant change in PD-L1 expression, the combination of RAPA + Honokiol markedly down-regulates the basal PD-L1 on renal cancer cells; and importantly, this combination treatment completely blocks c-Met-induced PD-L1 over-expression. Interestingly, although PD-L1 is down-regulated, the expression of MHC1 is unaltered following RAPA + Honokiol combination treatment. This observation suggests that in addition to preventing immune escape, this novel combination therapy may also promote the immune recognition of renal cancer cells by the cytotoxic T cells through MHC1 [38]. Our findings indeed indicate that the RAPA + Honokiol combination treatment promotes immune-mediated apoptosis of renal cancer cells.

As mentioned above, RAPA is used for the treatment of renal cancer, and it is also a good treatment option for the transplant patients to prolong their allograft survival; however, it has many limitations. Our results suggest that the combination therapy can have great potential to overcome the limitation of RAPA treatment, and also to maintain the immune suppression. The detailed mechanism of action for RAPA + Honokiol combination therapy still needs to be explored. PI-3K-Akt-mTOR pathway has significant cross-talk with other signaling molecules [39]. The oncogenic Ras is highly active in renal cancer cells primarily through the upstream receptor tyrosine kinases (like, c-Met); and it has critical cross-talk with the PI-3K-Akt pathway. Thus, when prolonged RAPA treatment activates Akt, the Ras pathway can become hyperactive, even in the absence of signals from receptor tyrosine kinases. Honokiol has a unique property to suppress Ras (in addition to its anti-inflammatory properties); and thereby can overcome the limitation of RAPA treatment alone.

In our previous studies, we have checked the individual effect of either Honokiol [13] or RAPA [40] in murine models of tumor growth; and we observed that the doses used for Honokiol/RAPA were well tolerated in mice, with no significant secondary effects. However, the efficiency and physiological significance of the combination therapy (RAPA + Honokiol) in restricting renal tumor growth needs to be tested in vivo. In our in vitro study, the effect of RAPA + HNK combination treatment was found to be synergistic as the CI (Combination Index) values were less than 1 (Supplementary Figure S5B). However, our goal was not to see a synergistic/additive effect; rather, we wanted to check if this combination therapy could overcome the limitation of RAPA treatment used in transplant patients.

Together, our observations suggest a novel combination therapy using RAPA + Honokiol that can effectively inhibit c-Met-induced and Ras-mTOR-mediated pathway(s) to restrict renal cancer growth as well as the immune escape of tumor cells. It can overcome the limitation of RAPA treatment and utilize its beneficial effects. Also, this combination treatment has significant therapeutic importance for the prevention of post-transplantation renal cancer. Due to the anti-inflammatory properties of Honokiol (in addition to its anti-tumorigenic function), it can be utilized for the treatment of transplant patients to prevent allograft rejection.

## 4. Materials and Methods

### 4.1. Reagents and Antibodies

Honokiol (HNK) and Rapamycin (RAPA) were purchased from Selleckchem. For Western blot analysis, primary antibodies against CDK2, CDK4, CDK6, Cyclin D1, p21, Bcl-2, Bcl-xL, c-Met, phospho-c-Met, HO-1, Akt, mTOR and S6 and species-specific secondary antibodies were obtained from Cell Signaling. Antibody against β-actin was purchased from Sigma Aldrich. Anti-PD-L1, phospho-Akt, phospho-S6 and phospho-mTOR antibodies were purchased from Thermo Fisher Scientific. Recombinant human hepatocyte growth factor (HGF) was purchased from Peprotech. 

### 4.2. Cell Lines

Human renal cancer cell lines (786-0 and ACHN) and mouse renal cancer cell line RENCA were obtained from American Type Culture Collection (ATCC). 786-0 and RENCA cell lines were maintained in RPMI 1640 medium. ACHN cells were maintained in EMEM medium. All medium were supplemented with 10% fetal bovine serum (FBS) and 1% penicillin-streptomycin antibiotics (Invitrogen, Carlsbad, USA). Normal renal proximal tubular epithelial cell (RPTEC) was obtained from Lonza, and was grown in renal epithelial cell basal medium with SingleQuots growth supplements (Lonza, Portsmouth, USA).

### 4.3. Generation of CRISPR/Cas9-Mediated c-Met Knock-out Cells

Renal cancer cells (786-0) were co-transduced with lentiviral particles encoding Cas9 and gRNA targeting either c-Met or control (ThermoFisher, Waltham, USA). Following antibiotic selection, stable clones were screened through Western blot to check the expression levels of total Met and HGF-induced phospho-Met. Clones with confirmed c-Met knock-out were selected and utilized.

### 4.4. Cell Proliferation Assay

Briefly, 10,000 cells/well were seeded in 96 well plate. Following treatments (as described), media was removed, and 10 μL of 5 mg/mL MTT (3-(4,5-Dimethylthiazol-2-yl)-2,5-Diphenyltetrazolium Bromide) dye (dissolved in phosphate buffered saline) was added to each well. Cell proliferation assay was performed using the MTT kit according to manufacturer’s protocol (Thermo Fisher Scientific). Briefly, plates were incubated in dark for 4 h; and after incubation formazon crystals were dissolved in DMSO (Dimethyl Sulfoxide) and absorbance was measured at 570 nm in a plate reader, and corrected against blanks, which consisted of culture medium processed in the same way as above in the absence of cells.

### 4.5. Cell Cycle Analysis

In total, 1 × 10^5^ cells were seeded in each well of 6 well tissue culture plates; and cells were allowed to attach and grow overnight. Cells were then serum starved for 24 h to synchronize them in Gap-1 phase. Following treatments, cells were collected through trypsinization, and stained with Propidium Iodide, and flow cytometry was performed on a FACSCalibur to assess the percent of cells in different phases of cell cycle.

### 4.6. Apoptosis Assay

Briefly, 1 × 10^5^ cells were seeded in each well of 6 well tissue culture plates and allowed to attach and grow overnight. Following treatments, the cells were collected through trypsinization; and they were stained using an allophycocyanin (APC)/FITC (Fluorescein isothiocyanate)-conjugated apoptosis detection kit (Thermo Fisher Scientific) according to manufacturer’s protocol. Cells were then analyzed by flow cytometry on a FACSCalibur. 

### 4.7. RENCA/Splenocytes Co-Culture

Splenocytes were isolated from the spleens of BALB/c mice following standard protocol. Briefly, extracted spleen was minced under aseptic condition in RPMI 1640 medium containing 10% FBS. Large tissues in the suspension were removed using 40 μm nylon mesh cell strainer and splenocytes were collected by centrifugation. Pellets were resuspended in fresh RPMI 1640 medium and then utilized in co-culture experiments. For co-culture experiment, isolated splenocytes (1 × 10^6^ cells) were added to the culture of RENCA cells grown in 6-well tissue culture plates. Following 72 h of incubation, supernatant containing splenocytes and unattached dead RENCA cells was removed. The adherent RENCA were collected by trypsinization and subjected to apoptosis assay as described earlier.

### 4.8. Measurement of Active/GTP-Bound Ras

The Active Ras Pull-Down and Detection Kit (Thermo Fisher Scientific) was used to measure the active-GTP-bound form of Ras in the cell lysate. This kit utilizes specific Ras-binding domain (RBD) of Raf-1 that can bind active GTP-bound form of Ras. The cells were lysed and incubated with GST-Raf-1-RBD and glutathione resin. The eluted samples were subjected to SDS (Sodium dodecyl sulfate)-polyacrylamide gel electrophoresis, transferred to a polyvinylidene difluoride (PVDF) membrane, and probed with anti-Ras antibody.

### 4.9. Reactive Oxygen Species (ROS) Detection

Briefly, 1 × 10^5^ cells were seeded in each well of 6 well tissue culture plates; and allowed to attach and grow overnight. Following treatments (as described), the cells were collected through trypsinization. Cellular ROS was detected utilizing a kit obtained from Enzo Life Sciences, New York, USA, and following manufacturer’s protocol.

### 4.10. Analysis of Proteins through Flow Cytometry

In total, 1 × 10^5^ cells were seeded in each well of 6 well tissue culture plates; and allowed to attach and grow overnight. Following treatments (as described), the cells were trypsinized, washed, and resuspended in phosphate-buffered saline containing 2% FBS. Cells (either permeabilized or non-permeabilized) were stained with respective FACS antibodies. Following incubation and washing, stained cells were analyzed by flow cytometry.

### 4.11. Western Blot Analysis

Following treatments (as described), the cells were lysed and protein samples were resolved on SDS-polyacrylamide gels and transferred to a polyvinylidene difluoride membrane (Millipore corporation, Burlington, USA). The membranes were incubated with primary antibodies and subsequently incubated with peroxidase-linked secondary antibodies. Reactive bands were detected using chemiluminescent substrate (ThermoFisher) on X-ray films.

### 4.12. Statistical Analysis 

Statistical significance was determined by Student’s *t* test. Differences with *p* < 0.05 were considered statistically significant. For combinatorial index (CI) compusyn software was utilized [41]. CI values of less than 1 were considered synergistic, while equal to 1 were considered as additive.

## 5. Conclusions

Although the mTOR inhibitor Rapamycin (RAPA) has anti-tumorigenic properties, it has many limitations. On the other hand, the natural product Honokiol (HNK) has both anti-tumorigenic and anti-inflammatory properties. This study suggests the importance of a novel combination therapy using HNK and RAPA to restrict c-Met-induced growth and immune escape of renal cancer cells, and overcome the limitation of RAPA treatment. In addition, this therapeutic combination has great potential to prevent the development of renal cancer in transplant patients. Together, RAPA + HNK combination can act as novel therapeutics for the treatment of renal cancer, with importance to post-transplantation cancer.

## Supplementary Materials

The following are available online at https://www.mdpi.com/2072-6694/12/7/1782/s1, Figure S1A: Effects of combinations of RAPA and HNK on phosphorylations of Akt, mTOR and S6, Figure S1B: HNK + RAPA combination treatment down-regulates HGF-induced phosphorylation of Akt, mTOR and S6 protein, Figure S2: Effects of HNK and RAPA on cell proliferation, Ras activation, and regulating cell cycle markers, Figure S3: Effects of RAPA + HNK combination on the apoptosis of ACHN and RPTEC, Figure S4: Effect of HNK and RAPA on HGF-mediated anti-apoptotic signals in ACHN renal cancer cells, and Figure S5: Effects of combination treatment with Rapamycin (RAPA) and Honokiol (HNK) against renal cancer cells.

## References

[1] Makhov P., Joshi S., Ghatalia P., Kutikov A., Uzzo R.G., Kolenko V.M. (2018). Resistance to Systemic Therapies in Clear Cell Renal Cell Carcinoma: Mechanisms and Management Strategies. Mol. Cancer Ther..

[2] Siska P.J., Beckermann K.E., Rathmell W.K., Haake S.M. (2017). Strategies to overcome therapeutic resistance in renal cell carcinoma. Urol. Oncol..

[3] Choueiri T.K., Motzer R.J. (2017). Systemic Therapy for Metastatic Renal-Cell Carcinoma. N. Engl. J. Med..

[4] Wells J.C., Graham J., Beuselinck B., Bjarnason G.A., Donskov F., Hansen A.R., McKay R.R., Vaishampayan U., De Velasco G., Duh M.S. (2019). Clinical Outcomes of First-line Sunitinib Followed by Immuno-oncology Checkpoint Inhibitors in Patients With Metastatic Renal Cell Carcinoma. Clin. Genitourin. Cancer.

[5] Gallagher M.P., Kelly P.J., Jardine M., Perkovic V., Cass A., Craig J.C., Eris J., Webster A.C. (2010). Long-term cancer risk of immunosuppressive regimens after kidney transplantation. J. Am. Soc. Nephrol..

[6] Balan M., Chakraborty S., Pal S. (2019). Signaling Molecules in Posttransplantation Cancer. Clin. Lab. Med..

[7] Sprangers B., Nair V., Launay-Vacher V., Riella L.V., Jhaveri K.D. (2018). Risk factors associated with post-kidney transplant malignancies: An article from the Cancer-Kidney International Network. Clin. Kidney J..

[8] Engels E.A., Pfeiffer R.M., Fraumeni J.F., Kasiske B.L., Israni A.K., Snyder J.J., Wolfe R.A., Goodrich N.P., Bayakly A.R., Clarke C.A. (2011). Spectrum of cancer risk among US solid organ transplant recipients. JAMA.

[9] van de Wetering J., Roodnat J.I., Hemke A.C., Hoitsma A.J., Weimar W. (2010). Patient survival after the diagnosis of cancer in renal transplant recipients: A nested case-control study. Transplantation.

[10] Karami S., Yanik E.L., Moore L.E., Pfeiffer R.M., Copeland G., Gonsalves L., Hernandez B.Y., Lynch C.F., Pawlish K., Engels E.A. (2016). Risk of Renal Cell Carcinoma Among Kidney Transplant Recipients in the United States. Am. J. Transplant..

[11] Dantal J., Soulillou J.P. (2005). Immunosuppressive drugs and the risk of cancer after organ transplantation. N. Engl. J. Med..

[12] Frasca G.M., Sandrini S., Cosmai L., Porta C., Asch W., Santoni M., Salviani C., D′Errico A., Malvi D., Balestra E. (2015). Renal cancer in kidney transplanted patients. J. Nephrol..

[13] Balan M., Chakraborty S., Flynn E., Zurakowski D., Pal S. (2017). Honokiol inhibits c-Met-HO-1 tumor-promoting pathway and its cross-talk with calcineurin inhibitor-mediated renal cancer growth. Sci. Rep..

[14] Balan M., Mier y Teran E., Waaga-Gasser A.M., Gasser M., Choueiri T.K., Freeman G., Pal S. (2015). Novel roles of c-Met in the survival of renal cancer cells through the regulation of HO-1 and PD-L1 expression. J. Biol. Chem..

[15] Augustine J.J., Bodziak K.A., Hricik D.E. (2007). Use of sirolimus in solid organ transplantation. Drugs.

[16] Xue Q., Nagy J.A., Manseau E.J., Phung T.L., Dvorak H.F., Benjamin L.E. (2009). Rapamycin inhibition of the Akt/mTOR pathway blocks select stages of VEGF-A164-driven angiogenesis, in part by blocking S6Kinase. Arterioscler. Thromb. Vasc. Biol..

[17] Rozengurt E., Soares H.P., Sinnet-Smith J. (2014). Suppression of feedback loops mediated by PI3K/mTOR induces multiple overactivation of compensatory pathways: An unintended consequence leading to drug resistance. Mol. Cancer Ther..

[18] Carracedo A., Pandolfi P.P. (2008). The PTEN-PI3K pathway: Of feedbacks and cross-talks. Oncogene.

[19] Fried L.E., Arbiser J.L. (2009). Honokiol, a multifunctional antiangiogenic and antitumor agent. Antioxid. Redox Signal..

[20] Ma L., Chen J., Wang X., Liang X., Luo Y., Zhu W., Wang T., Peng M., Li S., Jie S. (2011). Structural modification of honokiol, a biphenyl occurring in *Magnolia officinalis*: The evaluation of honokiol analogues as inhibitors of angiogenesis and for their cytotoxicity and structure-activity relationship. J. Med. Chem..

[21] Arora S., Singh S., Piazza G.A., Contreras C.M., Panyam J., Singh A.P. (2012). Honokiol: A novel natural agent for cancer prevention and therapy. Curr. Mol. Med..

[22] Chakraborty S., Balan M., Flynn E., Zurakowski D., Choueiri T.K., Pal S. (2019). Activation of c-Met in cancer cells mediates growth-promoting signals against oxidative stress through Nrf2-HO-1. Oncogenesis.

[23] Huang K., Chen Y., Zhang R., Wu Y., Ma Y., Fang X., Shen S. (2018). Honokiol induces apoptosis and autophagy via the ROS/ERK1/2 signaling pathway in human osteosarcoma cells in vitro and in vivo. Cell Death Dis..

[24] Hsiao C.H., Yao C.J., Lai G.M., Lee L.M., Whang-Peng J., Shih P.H. (2019). Honokiol induces apoptotic cell death by oxidative burst and mitochondrial hyperpolarization of bladder cancer cells. Exp. Ther. Med..

[25] Pan J., Zhang Q., Liu Q., Komas S.M., Kalyanaraman B., Lubet R.A., Wang Y., You M. (2014). Honokiol inhibits lung tumorigenesis through inhibition of mitochondrial function. Cancer Prev. Res. (Phila.).

[26] Chao L.K., Liao P.C., Ho C.L., Wang E.I., Chuang C.C., Chiu H.W., Hung L.B., Hua K.F. (2010). Anti-inflammatory bioactivities of honokiol through inhibition of protein kinase C, mitogen-activated protein kinase, and the NF-kappaB pathway to reduce LPS-induced TNFalpha and NO expression. J. Agric. Food Chem..

[27] Banumathy G., Cairns P. (2010). Signaling pathways in renal cell carcinoma. Cancer Biol. Ther..

[28] Teppo H.R., Soini Y., Karihtala P. (2017). Reactive Oxygen Species-Mediated Mechanisms of Action of Targeted Cancer Therapy. Oxid. Med. Cell. Longev..

[29] Marona P., Gorka J., Kotlinowski J., Majka M., Jura J., Miekus K. (2019). C-Met as a Key Factor Responsible for Sustaining Undifferentiated Phenotype and Therapy Resistance in Renal Carcinomas. Cells.

[30] Ostrand-Rosenberg S., Horn L.A., Haile S.T. (2014). The programmed death-1 immune-suppressive pathway: Barrier to antitumor immunity. J. Immunol..

[31] Saunders R.N., Metcalfe M.S., Nicholson M.L. (2001). Rapamycin in transplantation: A review of the evidence. Kidney Int..

[32] Sabarwal A., Agarwal R., Singh R.P. (2017). Fisetin inhibits cellular proliferation and induces mitochondria-dependent apoptosis in human gastric cancer cells. Mol. Carcinog..

[33] Lu C.H., Chen S.H., Chang Y.S., Liu Y.W., Wu J.Y., Lim Y.P., Yu H.I., Lee Y.R. (2017). Honokiol, a potential therapeutic agent, induces cell cycle arrest and program cell death in vitro and in vivo in human thyroid cancer cells. Pharmacol. Res..

[34] Guo Y.B., Bao X.J., Xu S.B., Zhang X.D., Liu H.Y. (2015). Honokiol induces cell cycle arrest and apoptosis via p53 activation in H4 human neuroglioma cells. Int. J. Clin. Exp. Med..

[35] Hahm E.R., Arlotti J.A., Marynowski S.W., Singh S.V. (2008). Honokiol, a constituent of oriental medicinal herb magnolia officinalis, inhibits growth of PC-3 xenografts in vivo in association with apoptosis induction. Clin. Cancer Res..

[36] Loboda A., Damulewicz M., Pyza E., Jozkowicz A., Dulak J. (2016). Role of Nrf2/HO-1 system in development, oxidative stress response and diseases: An evolutionarily conserved mechanism. Cell. Mol. Life Sci..

[37] Thompson R.H., Gillett M.D., Cheville J.C., Lohse C.M., Dong H., Webster W.S., Krejci K.G., Lobo J.R., Sengupta S., Chen L. (2004). Costimulatory B7-H1 in renal cell carcinoma patients: Indicator of tumor aggressiveness and potential therapeutic target. Proc. Natl. Acad. Sci. USA.

[38] Garcia-Lora A., Algarra I., Garrido F. (2003). MHC class I antigens, immune surveillance, and tumor immune escape. J. Cell. Physiol..

[39] Conciatori F., Ciuffreda L., Bazzichetto C., Falcone I., Pilotto S., Bria E., Cognetti F., Milella M. (2018). mTOR Cross-Talk in Cancer and Potential for Combination Therapy. Cancers.

[40] Basu A., Liu T., Banerjee P., Flynn E., Zurakowski D., Datta D., Viklicky O., Gasser M., Waaga-Gasser A.M., Yang J. (2012). Effectiveness of a combination therapy using calcineurin inhibitor and mTOR inhibitor in preventing allograft rejection and post-transplantation renal cancer progression. Cancer Lett..

[41] Wang K., Zhang C., Bao J., Jia X., Liang Y., Wang X., Chen M., Su H., Li P., Wan J.B. (2016). Synergistic chemopreventive effects of curcumin and berberine on human breast cancer cells through induction of apoptosis and autophagic cell death. Sci. Rep..

