# Supplementary material for: A Novel Combination Treatment with Honokiol and Rapamycin Effectively Restricts c-Met-Induced Growth of Renal Cancer Cells, and also Inhibits the Expression of Tumor Cell PD-L1 Involved in Immune Escape
Source: Cancers (Basel). 2020 Jul 3;12(7):1782. doi: 10.3390/cancers12071782 (PMC7408055; doi:10.3390/cancers12071782)
Supplement: Supplementary file 1 [file cancers-12-01782-s001.pdf]

Supplementary Figure S1A

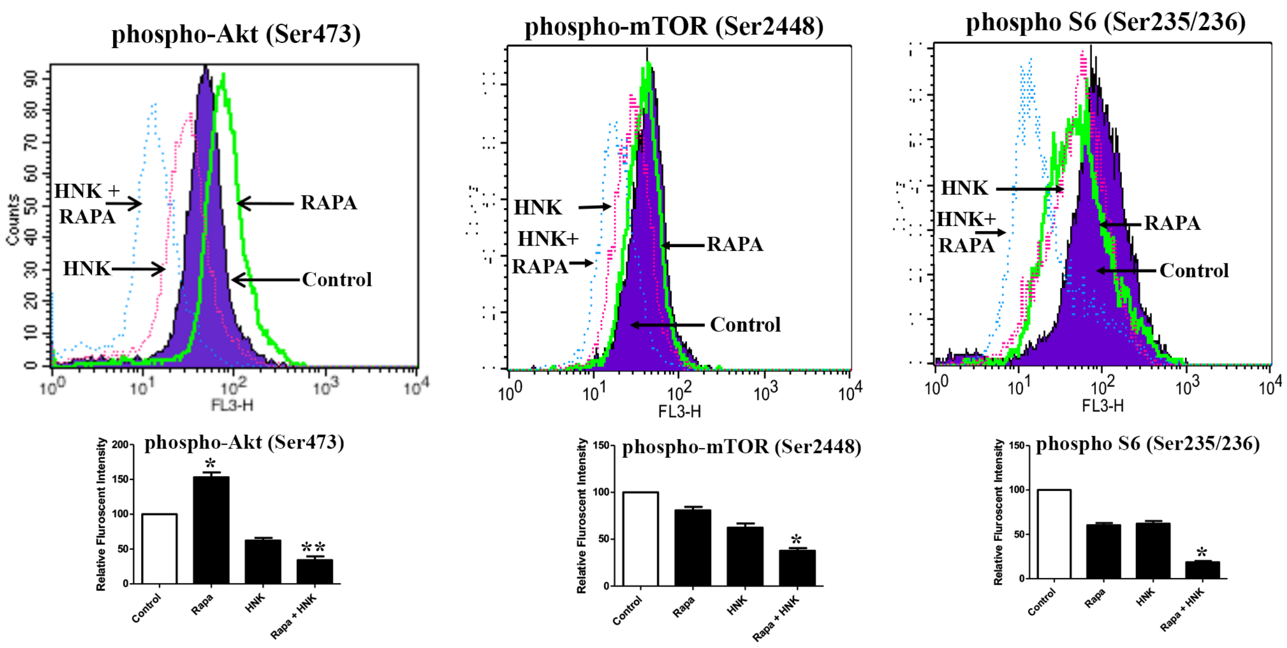

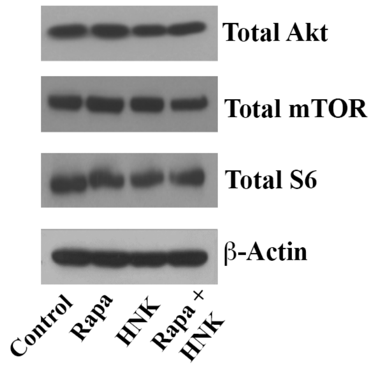

Effects of combinations of RAPA and HNK on phosphorylations of Akt, mTOR and S6: *Top histogram overlays*, serum starved 786-0 cells were treated with different combinations of RAPA (15 μM) and HNK (40 μM) for 4 hr. Following treatment, cells were analyzed for the intracellular levels of phospho-Akt (Ser473), phospho-mTOR (Ser2448) and phospho-S6 (Ser235/236) by flow cytometry. Data are representative data of three independent experiments and the bar graphs presented below histogram overlays represent the quantification of changes in relative fluorescence intensities when compared with the control (calculated as 100%). The *columns* represent the mean ± S.D of duplicate experimental readings of samples. *, $p < 0.05$ compared with control and **, $p < 0.05$ compared to HNK. *Bottom*, cell lysates, prepared from the samples as described above, were used to analyze the expression levels of Akt, mTOR and S6 proteins in Western blot. Data shown are representative of three independent experiments.

Supplementary Figure S1B

ACHN Cells

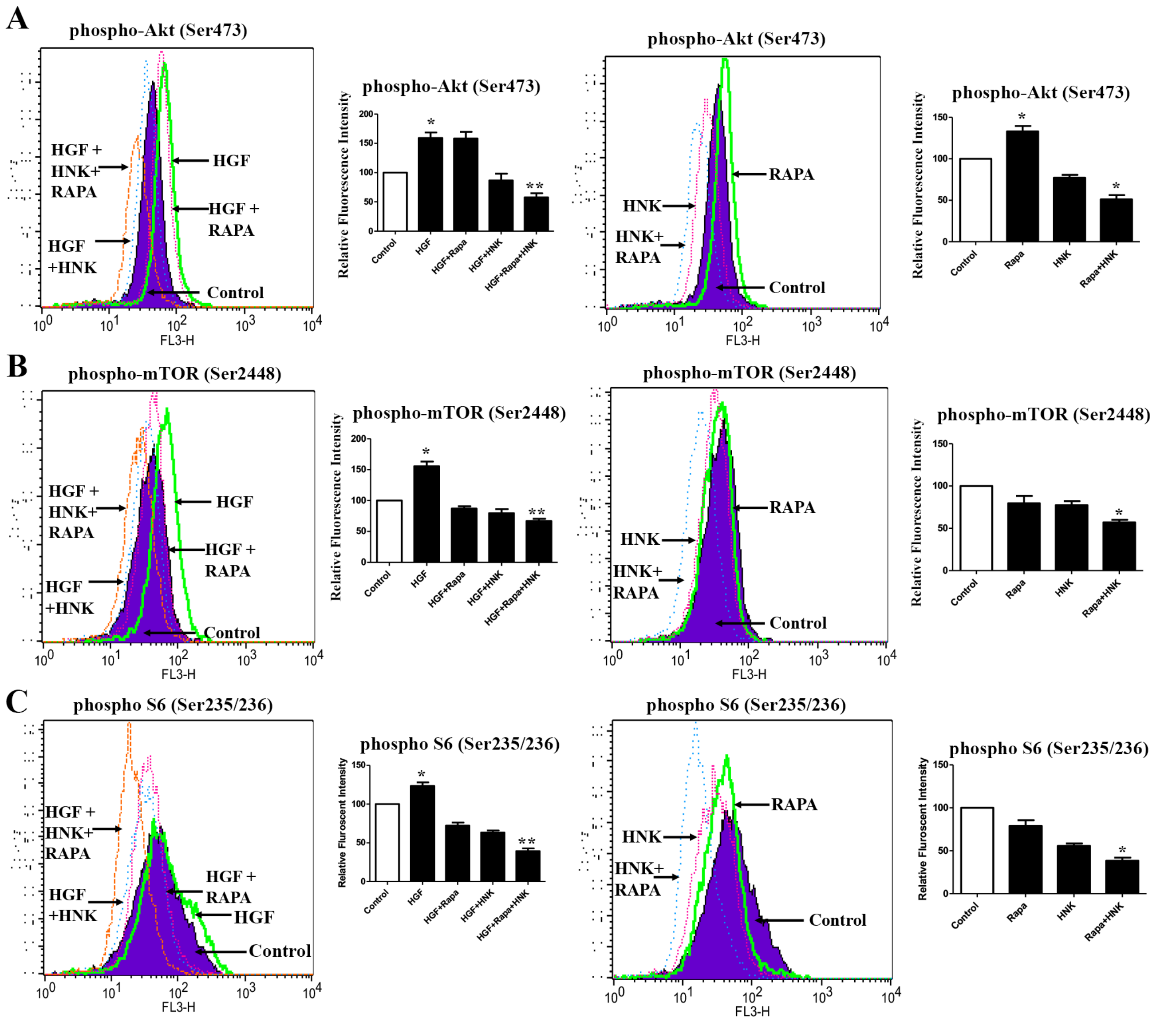

HNK + RAPA combination treatment down-regulates HGF-induced phosphorylation of Akt, mTOR and S6 protein: *A*, *B* and *C*, serum starved ACHN cells were pre-treated with different combinations of RAPA (15 μM) and HNK (40 μM) for 4 hr; and then incubated with either HGF (50 ng/ml) for 1 hr (left histogram overlays) or untreated (right histogram overlays). Vehicle-treated cells served as controls. Following treatment, cells were analyzed for the intracellular levels of phospho-Akt (Ser473), phospho-mTOR (Ser2448) and phospho-S6 (Ser235/236) by flow cytometry. *A-C*, representative data of three independent experiments and the bar graphs presented next to histogram overlays represent the quantification of changes in relative fluorescence intensities compared with the control (calculated as 100%). The *columns* represent the mean ± S.D of duplicate experimental readings. *, $p < 0.05$ compared with respective controls; **, $p < 0.05$ compared with HGF-treated cells.

Supplementary Figure S2

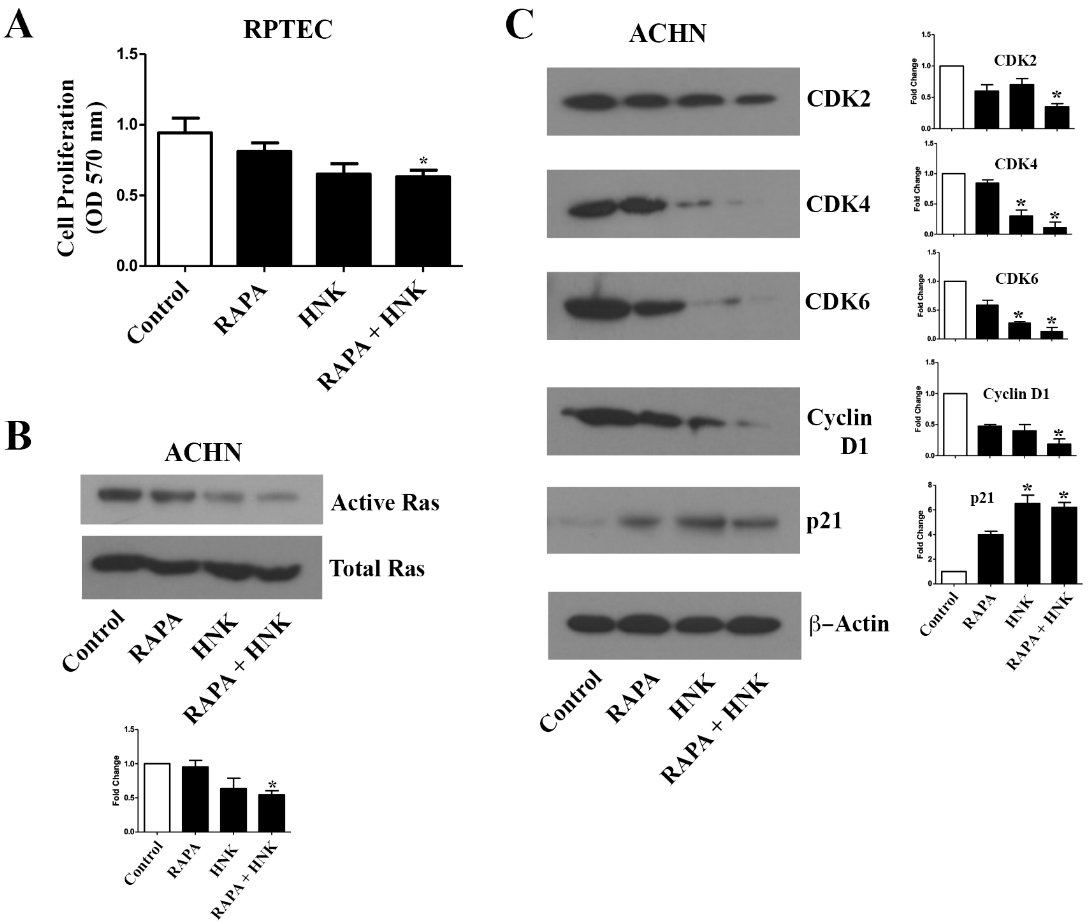

Effects of HNK and RAPA on cell proliferation, Ras activation, and regulating cell cycle markers:

A, RPTEC cells were treated with RAPA (15 μM) and Honokiol (40 μM) either alone or in combination for 48 hr and cell proliferation was measured by MTT assay. *B,* ACHN cells were treated with RAPA (15 μM) and Honokiol (40 μM) either alone or in combination for 1 hr. Following treatment, cell lysates were used to assess Ras activation statuses using GTP-bound Ras pull down assay kit, as described in "Materials and Methods" section. *C*, ACHN cells were treated with RAPA (15 μM) and Honokiol (40 μM) either alone or in combination for 48 hr. Following treatment, cell lysates were used to determine the expression levels of CDK2, CDK4, CDK6, Cyclin D1, p21 and β-actin by Western blot analysis; and the bar graphs presented next to the Western blots correspond to the fold changes in the expression of the indicated proteins, which were calculated by densitometric analysis of the intensities of protein bands normalized to those of β-actin. The control values were considered as 1 fold. *A - C*, results shown are representative of three independent experiments. The *columns* represent the mean ± S.D of duplicate experimental readings. *, $p < 0.05$ compared with respective controls.

Supplementary Figure S3

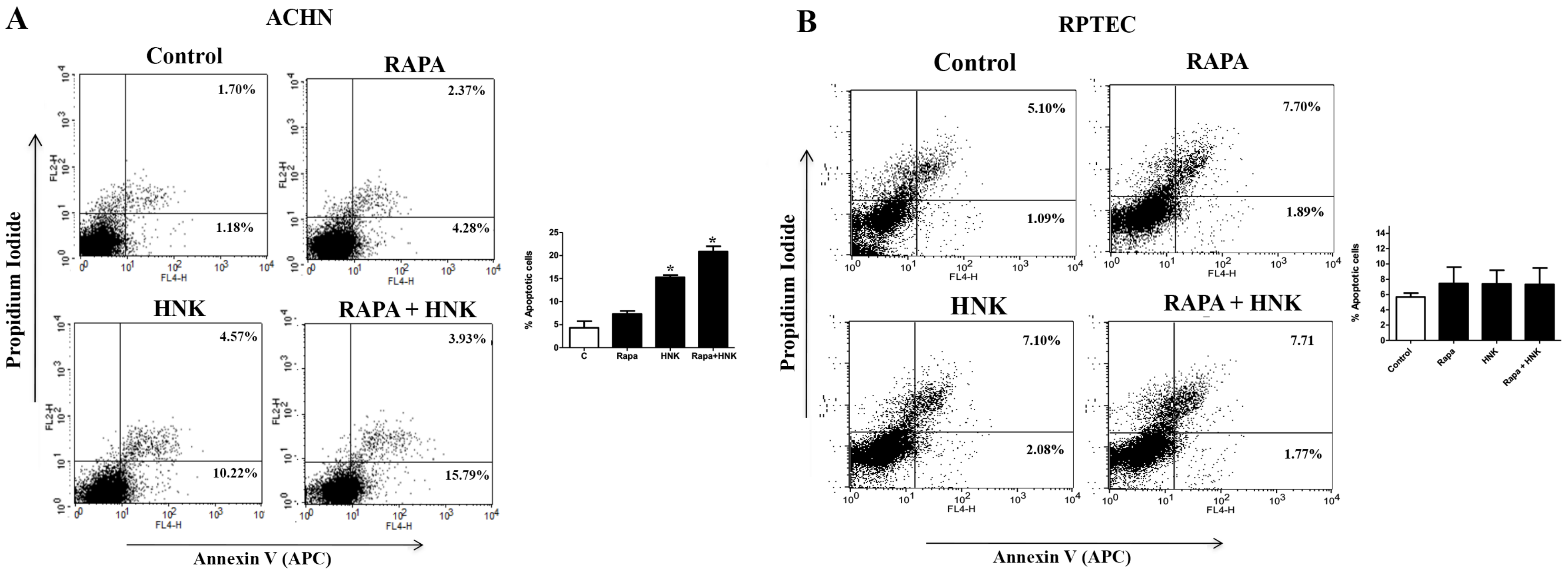

Effect of RAPA + HNK combination on the apoptosis of ACHN and RPTEC:

A and *B*, ACHN cells or RPTEC cells respectively, were treated with RAPA (15 µM) and Honokiol (40 µM) either alone or in combination for 48 hr. Following treatment, cells were stained with annexin V (APC) and propidium iodide and the apoptotic indices were analyzed by flow cytometry. Results shown are representative of three independent experiments. *A* and *B*, the bar graph presented next to the FACS analysis, represents the percentage of total (early + late) apoptotic cells. The *columns* represent the mean ± S.D of duplicate experimental readings.

*, $p < 0.05$ compared with respective controls.

Supplementary Figure S4

ACHN Cells

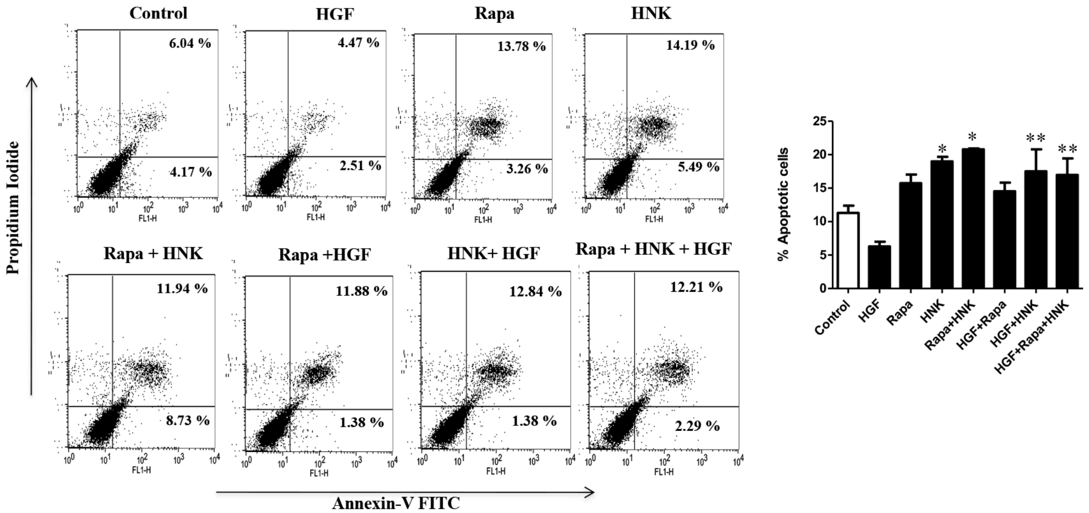

Effect of HNK and RAPA on HGF-mediated anti-apoptotic signals in ACHN renal cancer cells:

ACHN cells were treated with RAPA (15 uM) and Honokiol (HNK, 40 uM) either alone or in combination, and incubated in the presence or absence of HGF for 48 hr. Following treatment, cells were stained with annexin V (FITC) and propidium iodide, and apoptotic indices were analyzed by flow cytometry. The bar graph next to the FACS analysis represents the percentage of total (early + late) apoptotic cells. The columns represent the mean +/- S.D. of duplicate experimental readings. *, $p < 0.05$ compared with control and **, $p < 0.05$ compared with HGF-treated cells.

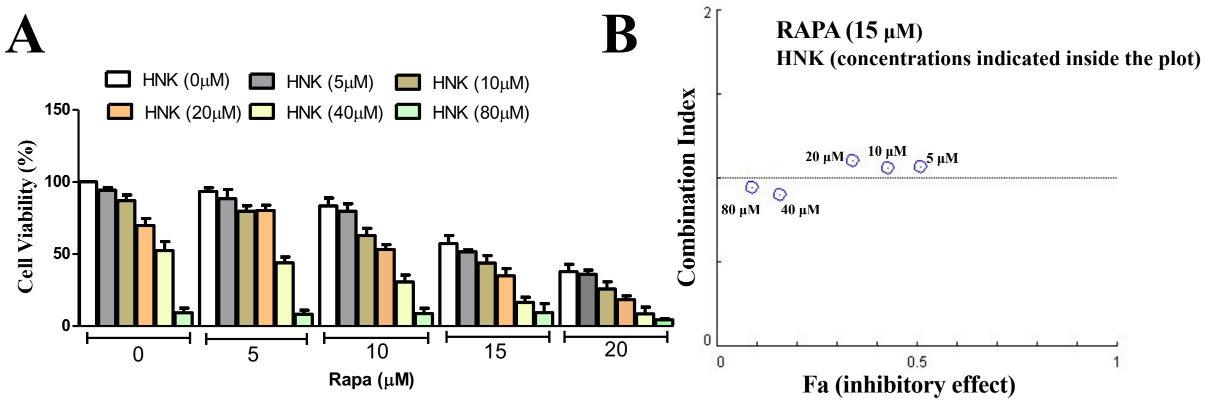

SUPPLEMENTARY FIGURE S5. **Effects of combination treatment with Rapamycin (RAPA) and Honokiol (HNK) against renal cancer cells:** *A*, 786-0 cells were treated with increasing doses of RAPA (5, 10, 15 and 20 μM) and Honokiol (5, 10, 20, 40 and 80 μM) either alone or in combination for 48 hr. Following treatment, cell proliferation was measured by MTT assay and the control values were considered 100%. The results showed that RAPA or HNK treatment resulted in a dose-dependent growth inhibition and co-treatment of RAPA and HNK significantly enhanced this effect. Treatment with either RAPA (15 μM) or HNK (40 μM) resulted in approximately 50% reduction in cell proliferation and these doses were used in all our experiments. *B*, to further examine the effects of RAPA+ HNK combination treatment, Combination Index (CI) data for RAPA (15 μM) and varying doses of HNK, and Fa ("Fraction Affected" or the inhibitory effect), analyzed by CompuSyn software, were plotted (Combination Index plot). Briefly, CI <1, = 1, and >1 represent synergistic, additive, and antagonistic effects, respectively. For the combination treatment with RAPA (15 μM) and HNK (40 μM), the CI values were less than 1, suggesting that RAPA and HNK may act synergistically in exerting the cytotoxic inhibitory effects on renal cancer cells. *A*, the *columns* represent the mean ± S.D of triplicate experimental readings. *B*, values represent mean ± S.D (n=2).
